# Secondary systemic artery to pulmonary artery and pulmonary vein fistulas following the video-assisted thoracic surgery for pneumothorax: a case report

**DOI:** 10.1186/s40792-017-0407-y

**Published:** 2018-01-02

**Authors:** Takuo Shimmyo, Takahiro Omori, Akira Hirano, Munetaka Masuda

**Affiliations:** 1 Department of Thoracic Surgery, Yokosuka General Hospital Uwamachi, 2-36 Uwamachi, Yokosuka City, Kanagawa 238-8567 Japan; 2 Department of Radiology, Yokosuka General Hospital Uwamachi, 2-36 Uwamachi, Yokosuka City, Kanagawa 238-8567 Japan; 3 Department of Surgery, Graduate School of Medicine, Yokohama City University, 3-9 Fukuura, Kanazawa-ku Yokohama City, Kanagawa 236-0004 Japan

**Keywords:** Secondary systemic artery to pulmonary vessel fistula, Video-assisted thoracic surgery, Pneumothorax, Embolization, Lung resection

## Abstract

**Background:**

The systemic artery to pulmonary vessel fistula (SAPVF) is a vascular anomaly characterized by penetration of nonbronchial systemic chest wall arteries into the lung parenchyma. To our knowledge, about 150 cases of SAPVF have been reported to date. Fifteen percent of SAPVF are congenital and occur in the presence of cardiopathy or pulmonary artery hypoplasia. Secondary SAPVF are caused by pleural adhesions that occur subsequent to inflammatory changes associated with conditions such as pleuritis, empyema, trauma, and surgery. Though several cases of secondary SAPVF as a post coronary artery bypass graft (CABG) complication have been reported, secondary SAPVF especially following video-assisted thoracic surgery (VATS) are relatively rare.

**Case presentation:**

A 19-year-old man was admitted to our hospital because of recurrence of left pneumothorax. His previous history included left and right pneumothorax at the ages of 15 and 16 years, respectively, which were treated by VATS. VATS was planned for the surgical indication of second postoperative recurrence. In the operation, the lingular segment with dilated pulsating pulmonary vessels adhered to the port scar of the chest wall, which was made at first VATS for pneumothorax. The computed tomography showed an abnormal connection between the branch of the systemic artery of the chest wall and the dilated pulmonary artery and pulmonary vein in the lingular segment. Left subclavian selective arteriography also showed hypertrophic blood vessels arose from the internal thoracic artery, the lateral thoracic artery, and the subscapular artery, which drained into the both the pulmonary artery and the pulmonary vein in the lingular segment. Despite of four sessions of embolization for aberrant arteries, the abnormal blood flow persisted. Partial resection of the left lingular segment was therefore performed. The patient has been disease-free about SAPVF for 2 years and 2 months after the last operation.

**Conclusions:**

We described our experience with a case of secondary SAPVF that was associated with fistulas between a systemic artery and both the pulmonary artery and pulmonary vein, which was developed after first VATS for pneumothorax. Radical resection was safely performed and effective after four sessions of embolization.

## Background

The systemic artery to pulmonary vessel fistula (SAPVF) is a vascular anomaly characterized by penetration of nonbronchial systemic chest wall arteries into the lung parenchyma, which was first reported by Burchell and Clagett in 1947 [1]. About 15% of SAPVF are congenital [2] and occur in the presence of cardiopathy or pulmonary artery hypoplasia [3]. Secondary SAPVF are caused by pleural adhesions that occur subsequent to inflammatory changes associated with conditions such as pleuritis, empyema, trauma, and surgery [1–5]. Though the several cases of secondary SAPVF as a post coronary artery bypass graft (CABG) complication have been reported [2], secondary SAPVF especially following video-assisted thoracic surgery (VATS) are relatively rare. We report a case of secondary systemic artery to pulmonary artery and pulmonary vein fistulas that developed after VATS for pneumothorax.

## Case presentation

**Fig. 1:**
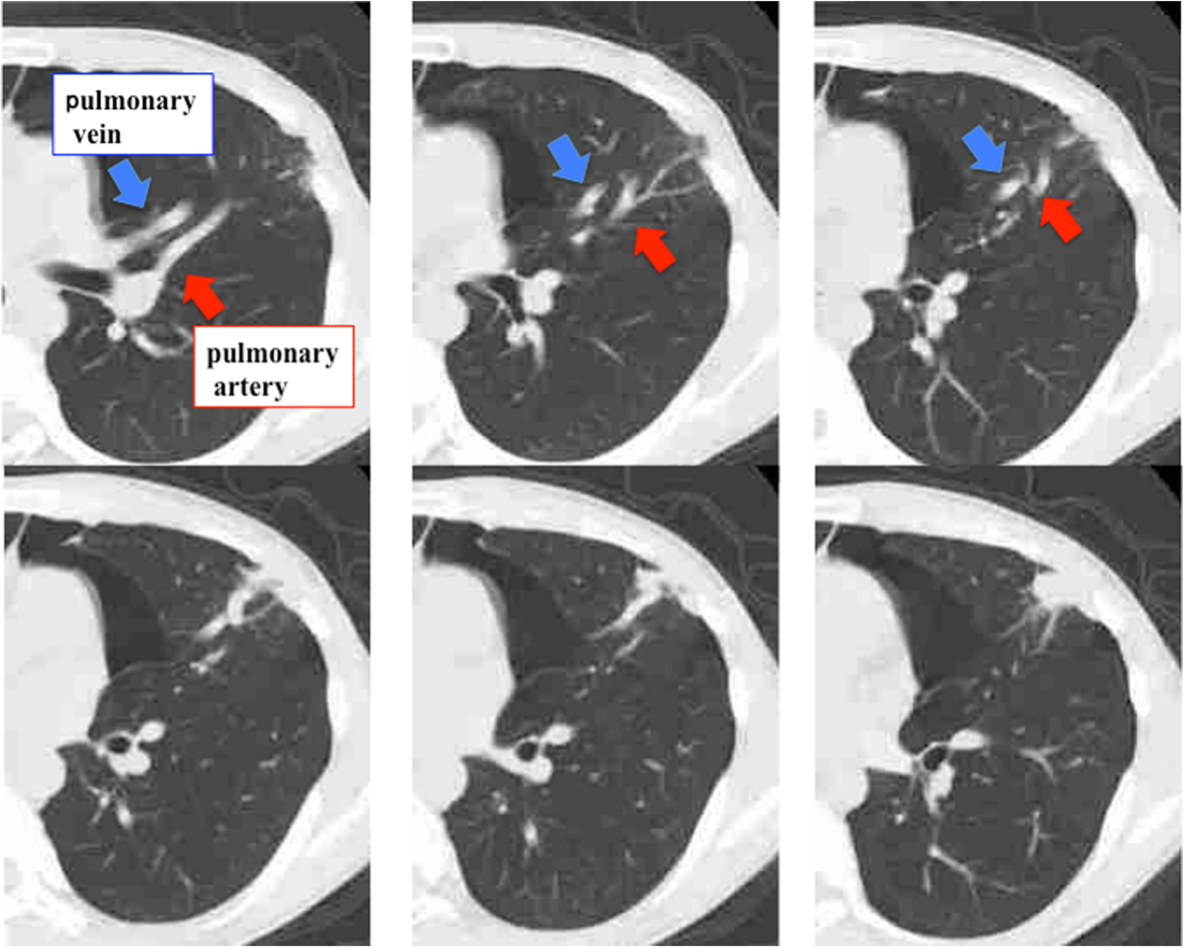
Preoperative non-enhanced chest CT scan findings. The abnormally dilated pulmonary artery and pulmonary vein in the lingular segment run towards the chest wall scar remaining at the site of surgical port placement during the previous operation

**Fig. 2:**
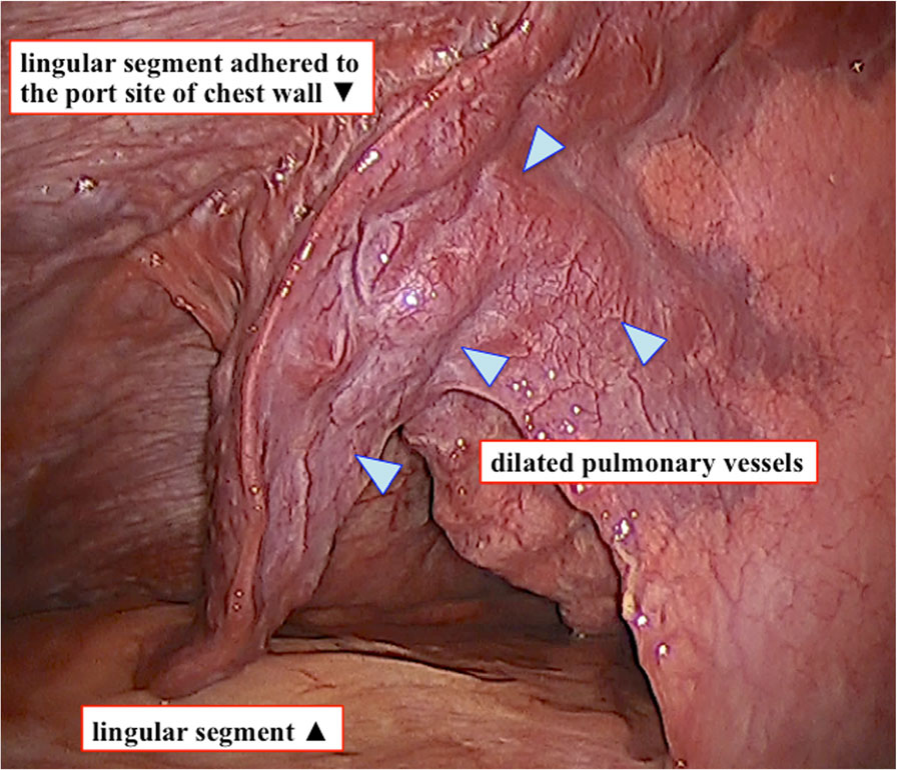
Intraoperative findings at second surgery for left pneumothorax. The lingular segment with dilated pulsating pulmonary vessels (arrow heads) adhered to the port site of chest wall, which was made at the first video-assisted thoracic surgery for pneumothorax

**Fig. 3:**
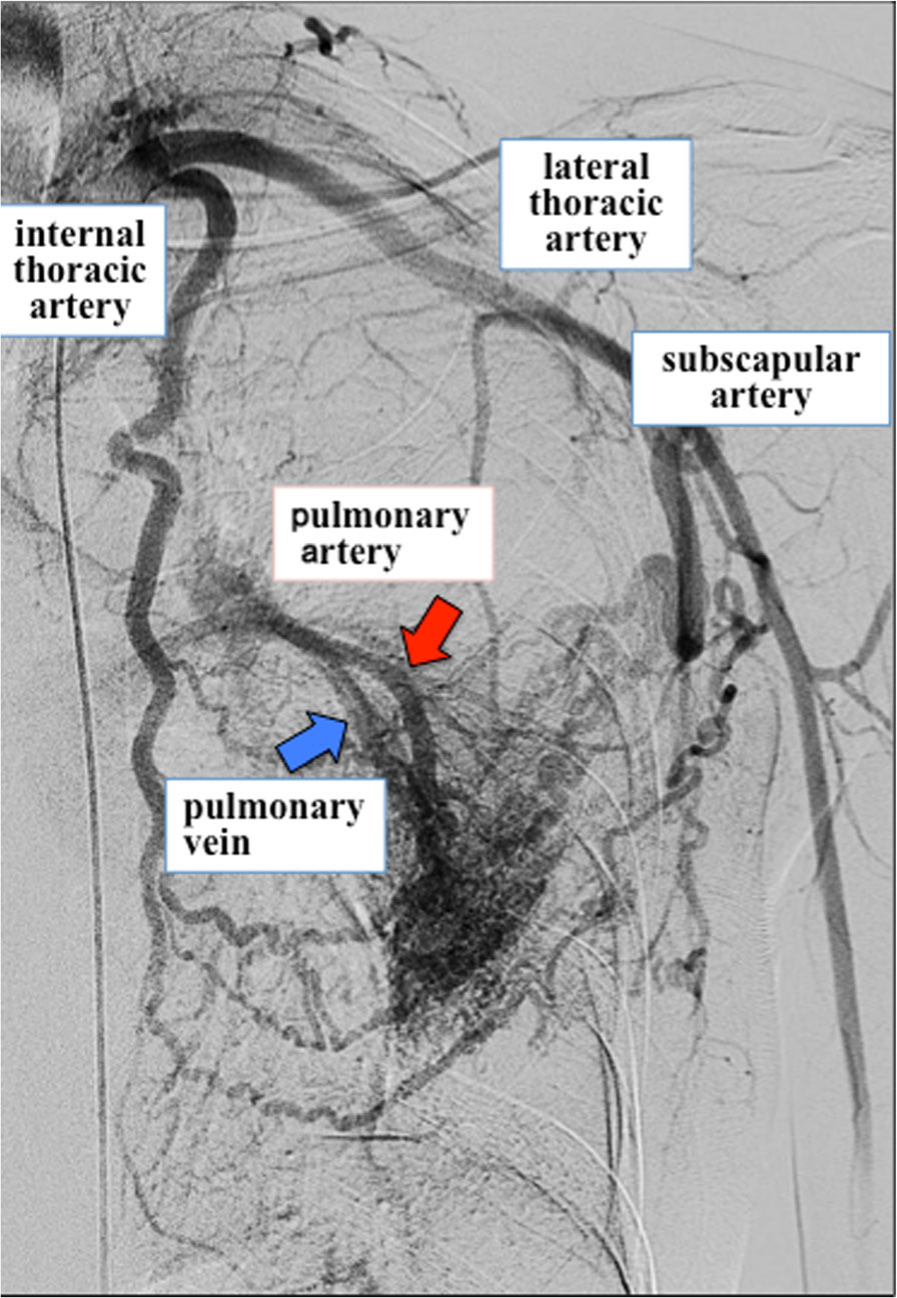
Left subclavian selective arteriogram findings. A left subclavian selective arteriogram, showing hypertrophic blood vessels that arose from the internal thoracic artery, the lateral thoracic artery, intercostal artery, and the subscapular artery arising from the axillary artery and drained into both the pulmonary artery (red arrow) and the pulmonary vein (blue arrow) in the lingular segment

A 19-year-old man was admitted to our hospital because of recurrence of left pneumothorax. His previous history included left and right pneumothorax at the ages of 15 and 16 years, respectively, which were treated by VATS. In the previous operation, simple resection of apical bullous lesion was carried out without surgical pleurodesis or covering any prosthetic sheets such as polyglycolic acid (PGA) sheets. Although the left lung inflated quite well and air leakage disappeared immediately after chest drainage, VATS was planned for the surgical indication of second postoperative recurrence. A preoperative non-enhanced computed tomography (CT) scan of the chest showed that the abnormally dilated pulmonary artery and pulmonary vein in the lingular segment ran towards the chest wall scar remaining at the surgical port site used at the previous operation (Fig. 1). In this operation, a small bullous lesion arising in segment 6 of the left lung was ligated, and the dilated pulsating pulmonary vessels at the periphery of the lingular segment adhered to the aforementioned chest wall scar remaining at the thoracoscopy port site that was previously made in the fourth intercostal space (Fig. 2). After this operation, we used contrast-enhanced CT scan for a suspected diagnosis of pulmonary vessel malformation. The examination revealed an abnormal connection between the branch of the systemic artery of the chest wall and the dilated pulmonary artery and pulmonary vein in the lingular segment. Left subclavian selective arteriography also showed hypertrophic blood vessels that arose from the internal thoracic artery, the lateral thoracic artery, and the subscapular artery arising from the axillary artery, which drained into the both the pulmonary artery and the pulmonary vein in the lingular segment (Fig. 3). Because the chest CT scan obtained at the first episode of left pneumothorax showed no parietopulmonary fistula, a secondary SAPVF caused by first VATS for the pneumothorax was diagnosed.

The patient had no symptoms after the cure of pneumothorax, but the vascular abnormalities might lead to shunt-induced pulmonary hypertension, heart failure, hemoptysis, and possibly rupture. Embolization of the aberrant arteries was therefore performed. Despite of four sessions of embolization, the abnormal blood flow slightly persisted. Partial resection of the left lingular segment was therefore performed 2 weeks after the last session of embolization to avoid recanalization and further neovascularization. The operation was carried out by dissection of the affected lung firstly at proximal side by a surgical stapler, and next, the adhesiolysis was safely done at the distal side of the affected lung by using energy devices such as ultrasonic scalpel and vessel-sealing device without any major bleeding. After the lung resection, the staple line was firstly covered with polyglycolic acid (PGA) sheets (NEOVEIL® Gunze, Tokyo, Japan) to prevent pulmonary fistula and bleeding followed by fibrin glue dripping (Bolheal® Kaketsuken, Kumamoto, Japan), and chest wall side was also covered by same methods. In addition, both staple line and chest wall side was finally covered with an oxidized regenerated cellulose sheet (SURGICEL® Ethicon, Somerville, NJ, USA) to prevent re-adhesion. The patient has been disease-free (both pneumothorax and SAPVF) for 2 years and 2 months after the last operation.

### Discussion

The most common abnormalities of the pulmonary vessels are arteriovenous malformation (AVM) or the racemose hemangioma of bronchial artery, while SAPVF is relatively rare. SAPVF was first reported by Burchell and Clagett in 1947 [1]. To our knowledge, about 150 cases of SAPVF have been reported to date [1–8]. Secondary SAPVF are caused by pleural adhesions that occur subsequent to inflammatory changes associated with conditions such as pleuritis, empyema, trauma, and surgery. Jabber et al. described in a systematic review about internal thoracic artery to pulmonary vasculature fistula that the 59% of all fistula cases were found after CABG surgery. On the other hand, the case of secondary SAPVF following VATS like our case were just a few [2]. Traumatic change from dissection of the internal thoracic artery as a bypass graft may lead to internal thoracic artery to pulmonary vasculature fistula as Jabbar’s report [2]. Several other reports also described the secondary SAPVF usually developed as a consequence of inflammatory processes of the pleura or lung or after blunt, open chest trauma or thoracotomy [1–8]. Interestingly, our case developed SAPVF regardless of the previous operation carried out without thoracotomy and without using any prosthetic sheets causing adhesion.

Most cases of SAPVF are unassociated with any symptoms, while SAPVF associated with severe hemoptysis, dyspnea due to cardiac failure, pulmonary hypertension, endocarditis, and chronic chest pain has occasionally been reported [8, 9]. In the past, the presence of a thoracic murmur or dyspnea provided clues to the diagnosis of SAPVF [3, 4], but recently, SAPVF has often been detected on the basis of abnormal findings such as increased lung vascularization or parenchymal infiltration on routinely obtained chest X-ray films or CT scans [5, 7]. The differential diagnoses include intrapulmonary AVM or pulmonary sequestration. However, these vascular abnormalities can be distinguished by angiography. The most common aberrant arteries in SAPVF are the internal thoracic arteries. The intercostal, axillary, diaphragmatic, and subclavian arteries can also be involved. In our patient, the intercostal artery, internal thoracic artery, lateral thoracic artery, and subscapular artery arising from the axillary artery drained into both the pulmonary artery and pulmonary vein in the lingular segment of the left lung.

Generally, SAPVF can be managed by embolization, surgical resection, or sometimes observation [3–6]. Embolization can be more effective in patients with a single or a few aberrant arteries than in those with multiple aberrant arteries. Our patient had many aberrant arteries supplied from the chest wall, which may be the reason for having to perform embolization four times. In patients such as ours who have many aberrant arteries, surgery is the treatment of choice to cure and prevent recurrence of SAPVF. However, some fistulas have an abundant blood flow. In such patients, preoperative embolization is recommended to reduce the risk of intraoperative blood loss. In our patient, although abnormal blood flow remained after embolization, surgical resection could be safely performed without any complications. Another consideration at operation is whether to use a prosthetic sheet to cover the post-resectional surface and thereby prevent re-adhesion and the recurrence of SAPVF. The optimal type of prosthetic sheet remains controversial. The oxidized regenerated cellulose sheet (SURGICEL®) shows to be reasonably well-thickened with minimal or milder adhesion than PGA sheet [10]. So, we used a PGA sheet firstly to prevent bleeding from dilated pulmonary vessels and aberrant arteries in chest wall and finally used an oxidized regenerated cellulose sheet to prevent adhesion.

## Conclusions

In conclusion, we described our experience with a case of secondary SAPVF that was associated with fistulas between a systemic artery and both the pulmonary artery and pulmonary vein, which was developed after first video-assisted thoracic surgery for pneumothorax. Radical resection was safely performed and effective after four sessions of embolization.

## Abbreviations

AVM: Arteriovenous malformation
CABG: Coronary artery bypass graft
CT: Computed tomography
PGA: Polyglycolic acid
SAPVF: Systemic artery to pulmonary vessel fistula
VATS: Video-assisted thoracic surgery
